# Human papillomavirus genotypes detected in clinician-collected and self-collected specimens from women living in the Mississippi Delta

**DOI:** 10.1186/1471-2334-13-5

**Published:** 2013-01-07

**Authors:** Philip E Castle, Julia C Gage, Edward E Partridge, Alfio Rausa, Patti E Gravitt, Isabel C Scarinci

**Affiliations:** 1 Global Cancer Imitative, Chestertown, MD, USA; 2 Division of Cancer Epidemiology and Genetics, National Cancer Institute, National Institutes of Health, DHHS, Bethesda, MD, USA; 3 University of Alabama at Birmingham, Birmingham, AL, USA; 4 Mississippi State Department of Health, Jackson, MS, USA; 5 Department of Epidemiology, Johns Hopkins School of Public Health, Baltimore, MD, USA

**Keywords:** Human papillomavirus (HPV), Self-collection, Pap, Cervical intraepithelial neoplasia, Cervix

## Abstract

**Background:**

There are no data available on human papillomavirus (HPV) infections in women living in the Mississippi Delta, where cervical cancer incidence and mortality among African American women is among the highest in the United States. The aim of this analysis was to report the age-specific prevalence of HPV in this population.

**Methods:**

We recruited 443 women, 26–65 years of age, from the general population of women living in the Mississippi Delta to participate; 252 women had been screened for cervical cancer within the last 3 years while 191 had not. Women underwent a pelvic exam and had clinician-collected Pap sample taken for the routine cervical cancer screening by cytology. Women were asked to collect a self-collected specimen at home and return it to the clinic. Both specimens were tested for HPV genotypes.

**Results:**

Four hundred and six women (91.6%) had HPV genotyping results for the clinician-collected and self-collected specimens. The prevalence of carcinogenic HPV was 18.0% (95% CI: 14.4%-22.1%) for clinician-collected specimens and 26.8% (95% CI: 22.6%-31.4%) for self-collected specimens. The concordance for the detection of carcinogenic HPV between clinician-collected and self-collected specimens was only fair (kappa = 0.54). While the prevalence of carcinogenic HPV in either sample decreased sharply with increasing age (p_trend_< 0.01), the prevalence of non-carcinogenic HPV did not, especially the prevalence of HPV genotypes in the alpha 3/4/15 phylogenetic group.

**Conclusions:**

The prevalence of carcinogenic HPV in our sample of women living in the Mississippi Delta was greater than the prevalence reported in several other U.S. studies. The high carriage of HPV infection, along with lack of participation in cervical cancer screening by some women, may contribute to the high cervical cancer burden in the region.

## Background

Persistent cervical HPV infection by cancer-associated or carcinogenic HPV is the necessary cause of cervical cancer [1,2]. DNA testing for HPV is more sensitive but less specific for detection of precancerous lesions and early cancer than cervical cytology [3-5]. HPV DNA testing of self-collected specimens is equally sensitive as cytology [6] and could be used to reach populations not being screened by current programs [7-15].

The Mississippi Delta is a little studied population that has historically had several-fold higher annual rates of cervical cancer incidence and mortality than those for the general U.S. [16,17]. The overarching goal of our study in the Mississippi Delta was to study the acceptability of self-collection and HPV testing as an alternative to Pap testing. We previously reported that under-screened women were almost twice as likely to choose self-collection over free Pap testing, and twice as many of those who chose self-collection completed their screening compared those who chose free Pap testing [18]. The goal of this specific analysis was examine the impact of age and anatomic site of specimen collection on the prevalence of HPV, as a proxy for specificity. We wanted to understand the potential impact of using self-collection and HPV DNA testing on the patient.

## Methods

Recruitment and clinical methods were detailed previously [19]. Briefly, women undergoing routine screening (n = 252)(“screened”) or had not been screened in the last three years (n = 191) (“under-screened”) were recruited as part of a larger study of self-collection and HPV DNA testing in 4 counties (Tallahatchie, Leflore, Sunflower, and Washington) located in northwest Mississippi and part of the Mississippi Delta. Women aged 26 to 65 years of age, non-pregnant, with a cervix, and willing to provide written, informed consent were enrolled into the study. Institutional review boards from the NCI, UAB, State of Mississippi Health Department, and Westat approved this study.

For all participants, cervical specimens were collected into PreservCyt (Hologic, Bedford, MA, USA) for routine cervical cytology and the residual was retained for HPV testing. Women were given a kit for self-collection at home, including a self-collection device [20,21] (http://patft.uspto.gov/netacgi/nph-Parser?Sect1=PTO1&Sect2=HITOFF&d=PALL&p=1&u=%2Fnetahtml%2FPTO%2Fsrchnum.htm&r=1&f=G&l=50&s1=6,475,165.PN.&OS=PN/6,475,165&RS=PN/6,475,165) and a vial of Scope™ (Proctor and Gamble, Cincinnati, OH, USA) mouthwash for specimen transport [22]. The self-collection device physically and mechanically resembles a tampon and includes an outer sheath to shield the collection from vaginal contamination and irrelevant vaginal HPV infections. Women were instructed to insert the device into their anterior vagina, expose the Dacron collection tip to obtain a sample of cervical cells, retract the tip into the sheath, remove the device, and eject the tip into the transport medium. We used mouthwash as our transport medium to avoid giving kits containing toxic liquid-based cytology medium to our participants to take home [22].

Specimens were tested for 37 HPV genotypes (6, 11, 16, 18, 26, 31, 33, 35, 39, 40, 42, 45, 51–56, 58, 59, 61, 62, 64, 66–73, 81–84, 82v, and 89) using Linear Array (LA; Roche Molecular Systems, Pleasanton, CA, USA) [23,24]. HPV16, 18, 31, 33, 35, 39, 45, 51, 52, 56, 58, 59, and 68 were classified as the certain or probable carcinogenic HPV genotypes [25]. HPV genotypes were also classified according to broad branches in the phylogenetic tree for HPV genotypes [26,27]: 1) HPV genotypes 6, 11, 40, and 42, which are classified in the alpha 1, 8, and 10 group (alpha 1/8/10 genotypes). HPV 6 and 11 cause 90% of *condyloma accuminata* (genital warts); 2) HPV genotypes 16, 18, 26, 31, 33, 35, 39, 45, 51–56, 58, 59, 64, 66, 67, 68, 69, 70, 73, 82, and 82v, which are classified in the alpha 5, 6, 7, 9, and 11 group (alpha 5/6/7/9/11 genotypes). All the carcinogenic and borderline carcinogenic HPV genotypes are found in these species; and 3) HPV genotypes 61, 62, 71, 72, 81, 83, 84, and 89, which are classified in the alpha 3, 4, and 15 group (alpha 3/4/15 genotypes). There is some evidence that these HPV genotypes in these species have a predilection for vaginal tissue [28-31].

Logistic regression was used to calculate odds ratios (OR), 95% confidence intervals, and p values to test for differences between study groups (screened vs. under-screened) and linear trends with age groups (26–39, 30–39, 40–49, and 50–65 years) for testing HPV positive for categories of HPV. For categories of HPV prevalences, both crude and adjusted for study group and age group, were calculated. Binomial exact 95% confidence intervals were used where noted. An exact version of McNemar chi-square test or a symmetry chi-square test was used to test for differences in prevalence for individual or specific group of HPV genotypes from the same specimen or for same individual or specific group of HPV genotypes from different specimens.

A *post-hoc* power calculation based on a McNemar chi-square test indicated that 1) a sample size of 406 pairs for the whole analysis population achieves 80% power (alpha = 0.05) to detect a difference in prevalence of 4.5% between specimens collections if the discordance was 10%, of 6.4% if the discordance was 20%, and of 7.8% if the discordance was 30%, and 2) a sample size of 312 pairs for women 30 years and older achieves 80% power (alpha = 0.05) to detect a difference in prevalence of 5.2% between specimens collections if the discordance was 10%, of 7.3% if the discordance was 20%, and 8.9% if the discordance was 30%.

A p value of < 0.05 was considered statistically significant. STATA Version 11.1 was used for these analyses (StataCorp, College Station, Texas, USA).

## Results

There were HPV testing results available for 440 (99.7%) clinician-collected specimens and 409 (92.3%) self-collected specimens. The analyses were restricted to the 406 (91.6%) women for whom both results were available, which included 231 screened women and 175 under-screened women. Of the 406 subjects included in this analysis, 370 (91.1%) identified themselves as Black/African American, 33 (8.1%) as White/Caucasian, and 3 (0.7%) as other. The mean and median ages were 37.5 years and 36 years, respectively.

We used logistic regression to show the impact of the two study groups and age on detection of HPV. As shown in Table 1, there was no significant difference in the HPV detection between study populations for categories of any HPV, carcinogenic HPV, non-carcinogenic HPV, and three phylogenetically defined categories of alpha 1/8/10, alpha 5/6/7/9/11, and alpha 3/4/15 HPV genotypes between populations for clinician-collected specimens or for self-collected specimens. We noted that in general the under-screened population had more HPV, albeit not significantly so; underscreened women were non-significantly more likely to have alpha 1/8/10 genotypes compared to screened women (OR = 1.9, 95% CI: 0.74-4.7). Because there was no evidence of significant differences in the prevalence of HPV between the screened and under-screened groups, the two were combined henceforth.

**Table 1:** Results of logistic regression models to examine the relationship of study population and age on detection of HPV groups

	**Clinician collected**				**Self-collected**			
	**OR**	**p**	**95% CI**	**p**_**trend**_	**OR**	**p**	**95% CI**	**p**_**trend**_
Any HPV	1.2	0.4	0.77-1.8	0.01	1.0	0.9	0.68-1.6	0.03
Carcinogenic	1.1	0.6	0.66-2.0	<0.001	1.4	0.1	0.90-2.3	0.004
Non-Carcinogenic	1.1	0.7	0.69-1.8	0.8	0.73	0.2	0.46-1.2	0.7
alpha 1/8/10	1.4	0.6	0.44-4.5	0.5	1.9	0.2	0.74-4.7	0.8
alpha 5/6/7/9/11	0.94	0.8	0.59-4.5	0.003	1.1	0.6	0.74-1.7	0.01
alpha 3/4/15	1.1	0.8	0.64-1.8	0.4	0.79	0.3	0.51-1.2	0.7

Odds ratios (OR) with 95% confidence intervals (95%CI) were calculated as a measure of association of women not having been screened in the last 3 years (vs. the reference of those who had) with HPV detection; p value was included for reference. Age groups were modeled continuously to assess the age trend (ptrend).

Increasing age was statistically associated with a lower likelihood of testing positive of any HPV, carcinogenic HPV, and alpha 5/6/7/9/11 genotypes, but not for alpha 3/4/15 genotypes, for both specimen types (Table 1).

We calculated the prevalence of HPV categories by collection method, and the concordance between specimens, as shown in Table 2; prevalence adjusted for age group and the population group (screened vs. under-screened) was not appreciably different (data not shown). The prevalence of any HPV was 42.4% (95% CI: 37.5%-47.3%) for clinician-collected specimens and 54.7% (95% CI: 49.7%-59.6%) for self-collected specimens; the discordance (disagreement) was 27% and therefore the *post-hoc* power was 99.7%. The adjusted prevalence of carcinogenic HPV was 18.0% (95% CI: 14.4%-22.1%) for clinician-collected specimens and 26.8% (95% CI: 22.6%-31.4%) for self-collected specimens. Self-collected specimens were more likely to test positive for any HPV, carcinogenic HPV, non-carcinogenic HPV, alpha 5/6/7/9/11, and alpha 3/4/15 genotypes than clinician-collected specimens (p < 0.001). As a consequence of the differences in HPV detection by specimen type, the concordance between the two specimens for HPV detection in any HPV category was only fair, with kappa values around 0.50.

**Table 2:** Prevalence of HPV groups in clinician- and self-collected specimens (n = 406)

					**Prevalence**				**Concordance**			
	**Clinician-collected**		**Self-collected**			**% Positive agreement**			
	**Prevalence**	**95% CI**	**Prevalence**	**95% CI**	**% Agreement**		**Kappa**	**p**	
Any HPV	42.4%	37.5%-47.3%	54.7%	49.7%-59.6%	73%	57%	0.48	<0.001
Carcinogenic	18.0%	14.4%-22.1%	26.8%	22.6%-31.4%	84%	47%	0.54	<0.001
Non-Carcinogenic	31.8%	27.3%-36.5%	44.6%	39.7%-49.6%	75%	53%	0.49	<0.001
alpha 1/8/10	3.2%	1.7%-5.4%	5.2%	3.2%-7.8%	97%	42%	0.57	0.06
alpha 5/6/9/11	28.8%	24.5%-33.%	41.4%	36.5%-46.4%	77%	51%	0.51	<0.001
alpha 3/4/15	21.2%	17.3%-25.5%	31.8%	27.3%-36.5%	82%	48%	0.53	<0.001

Differences in prevalence detected in clinician- and self-collected specimens were tested for statistical differences using an exact version of McNemar’s chi-square test. P value of less than 0.05 is considered significant and indicated by bolded font.

The prevalence of carcinogenic HPV for women 30 and older (n = 312), the ages at which HPV and cytology cotesting is acceptable by national screening guidelines [32-34], was 14.4% (95% CI: 10.7%-18.8%) for clinician-collected specimens and 24.0% (95% CI: 19.4%-29.2%) for self-collected specimens, the latter of which was significantly greater than the former (p < 0.001). The discordance (disagreement) was 17% and therefore the *post-hoc* power was 98.7%.

The concordance for HPV detection, categorizing hierarchically according to cancer risk, for the two specimens is shown in Table 3. The crude kappa was 0.47 and the percentage agreement was 66%. There was a significant difference in detection of HPV classified in this manner (p = 0.0002), primarily because the following discordant (self-collected/clinician-collected) results were common: 1) non-carcinogenic HPV/HPV negative; 2) carcinogenic HPV/HPV negative; and 3) carcinogenic HPV/non-carcinogenic HPV.

**Table 3:** A comparison of detection of human papillomavirus (HPV) genotypes, classified hierarchically according to cancer risk (HPV16>HPV18>other carcinogenic HPV>non-carcinogenic HPV>HPV negative), for clinician-collected and self-collected specimens from women living in the Mississippi Delta

		**Self collected**					
		**HPV16**	**HPV18**	**Other Carc.**	**Non Carc.**	**HPV Negative**	**Total**
Clinician Collected							
HPV Negative	n	5	5	23	46	155	234
	% cell	1.2%	1.2%	5.7%	11.3%	38.2	
Non Carcinogenic	n	2	3	13	62	19	99
	% cell	0.5%	0.7%	3.2%	15.3%	4.7%	
Other Carcinogenic	n	1	2	44	4	7	58
	% cell	0%	1%	11%	1%	2%	
HPV18	n	0	3	1	0	1	5
	% cell	0.%	0.7%	0.2%	0.0%	0.2%	
HPV16	n	4	1	2	1	2	10
	% cell	1.0%	0.2%	0.5%	0.2%	0.5%	
Total		12	14	83	113	184	406

The number (n) and cell percentage (% cell) for each pairwise results is shown. The crude kappa was 0.47, percent agreement was 66%, and symmetry chi-square was p = 0.0002. Abbreviation: carc., carcinogenic.

The agreement statistics for detection of all 37 HPV genotypes individually in self-collected vs. clinician-collected specimens were a kappa value of 0.56 (95% CI: 0.52-0.61), the total agreement of 97.8%, and the positive agreement of 40.0%, with self-collected specimens more likely to test positive for any of the HPV genotypes than clinician-collected specimens (p < 0.0001). The prevalence for individual HPV genotypes is shown in the Additional file 1: Table S1. The 5 most common HPV genotypes found in clinician-collected specimens were HPV54 (4.9%), HPV62 (4.7%), HPV83 (4.4%), HPV52 (3.9%), and HPV71 (3.4%); prevalences of HPV16 and HPV18, the two genotypes targeted by the current generation of HPV vaccines were 2.5% and 1.5%, respectively. The 5 most common HPV genotypes found in self-collected specimens were HPV54 (8.1%), HPV83 (7.9%), HPV70 (7.4%), HPV62 (7.4%), and HPV81 (6.4%); the prevalences of HPV16 and HPV18, the two genotypes targeted by the current generation of HPV vaccines were 3.0% and 3.9%, respectively. Individually, HPV18, 33, 54, 55, 62, 68, 70, 81, 83, and 84 were more commonly detected (p < 0.05) in self-collected specimens than clinician-collected specimens.

**Figure 1:**
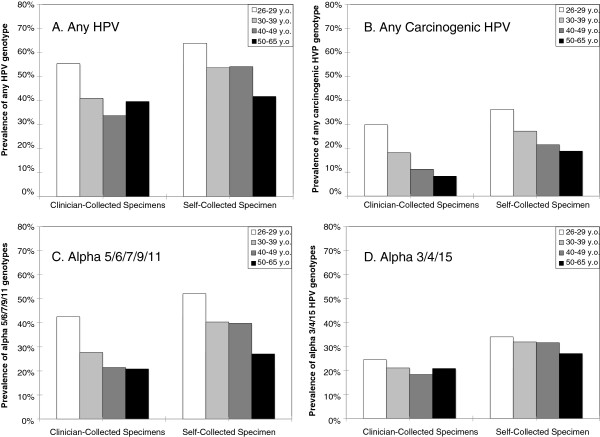
**The age group-specific prevalence of any (A), any carcinogenic (HPV16, 18, 31, 33, 35, 39, 45, 51, 52, 56, 58, 59, and 68) (B), any alpha 5/6/7/9/11 (HPV16, 18, 26, 31, 33, 35, 39, 45, 51-56, 58, 59, 64, 66, 67, 68, 69, 70, 73, 82, and 82v) (C), and alpha 3/4/15 (HPV 61, 62, 71, 72, 81, 83, 84, and 89) (D) human papillomavirus (HPV) types as detected in clinician-collected cervical specimens and self-collected cervicovaginal specimens.** Age groups were 26-29 (white bars), 30-39 (light gray bars) , 40-49 (medium gray bars), and 50-65 (black bars) years old (y.o.)

Shown in Figure 1 are the age group-specific patterns of prevalences for any HPV, any carcinogenic HPV, alpha 5/6/7/9/11 genotypes, and any alpha 3/4/15 genotypes for the two groups combined. While the prevalence of carcinogenic HPV detected in clinician-collected (p_trend_ = 0.0002) and self-collected (p_trend_ = 0.009) specimens decreased with increasing age, the prevalence of non-carcinogenic HPV did not (p_trend_ = 0.3 for clinician-collected, p_trend_ = 0.08 for self-collected). Grouping of HPV genotypes based on branches in the phylogenetic tree highlighted these differences in age trends. While the prevalence of alpha 5/6/7/9/11 detected in clinician-collected (p_trend_ = 0.001) and self-collected (p_trend_ = 0.007) decreased with increasing age, the prevalence of alpha 3/4/15 did not (p_trend_ = 0.4 for clinician-collected, p_trend_ = 0.4 for self-collected).

## Discussion

We found the prevalence of carcinogenic HPV to be relatively high in this population compared to other U.S. populations. The prevalence of carcinogenic HPV in clinician-collected specimens from women 30 and older in this population was approximately 2-fold higher than what has been observed at Kaiser Permanente Northern California (6.5% in women 30 and older) [35], and 40% higher in both a study that included women attending sexually transmitted infection (STI) clinics (~10% in women 30 and older) [36] and in a state-wide population study of women living in New Mexico (~10% in women 31 and older) [37], a state that ranks along with Mississippi as one of the poorest states in the US. The high prevalence of carcinogenic HPV reported in this study was not due to the use of LA for HPV detection as the prevalence of HPV was similar using Hybrid Capture 2 (p = 0.9) [19] the test used in these other reports in two of the aforementioned studies (35;36). We speculate that the higher prevalence of carcinogenic HPV, which has been shown to correlate with cervical cancer incidence [38], and a lack of screening in a subset of women living in the Mississippi Delta may in part account for the higher annual rate of cervical cancer incidence in this region compared to other places in the U.S. [16].

We note that this study was only cross-sectional and therefore could not assess the total lifetime exposure to HPV, nor could we measure HPV persistence, which precedes and predicts the development of cervical precancerous lesions [39]. Yet HPV prevalence has been correlated with the risk of cervical cancer [38], supporting the possibility that that this population of women living in the Mississippi Delta are at a higher risk than the general U.S. population.

The prevalence of HPV and the HPV genotypes detected was strongly influenced by both the age of the patient and the type of sampling (clinician vs. self). The higher prevalence of carcinogenic HPV in women under 30 (vs. 30 and older) suggests may preclude the use of self-collection and HPV testing in these younger women although the decision to use it has to be weighed against the possibility that these higher-risk women may not get the screening they need in the future.

While we did not observe an increase in alpha 3/4/15 genotypes in cervical specimens with age as observed in other studies (29;30), the prevalence of these HPV genotypes remained relatively unchanged with increasing age. As a consequence, the proportion of alpha 3/4/15 genotypes in the cervical specimens among any HPV infections increased significantly with age so that the prevalence of alpha 5/6/7/9/11 and alpha 3/4/15 genotypes were approximately the same in women 50 and older. To our surprise, the age pattern in the vagina as measured by self-collection was similar to that of the cervix, with higher prevalence of alpha 5/6/7/9/11 genotypes than alpha 3/4/15 genotypes in young women rather than the preponderance of alpha 3/4/15 genotypes at all ages. The differences in this study versus other studies could be due to the relative small sample size in this study or unmeasured differences in sexual behaviors. Another possibility is that self-collection device used in this study did a better job of sampling the cervix, making the self-collected specimens more representative of the cervical milieu of HPV genotypes than observed in other studies.

We note that the one of our limitations for this study is that we used non-FDA approved HPV test, self-collection device, and transport medium. Linear Array, the HPV test used in this study, is one of the standard methods for HPV genotyping [40] and has been shown to correlate well with Hybrid Capture 2 [24,41] and cobas4800 [42], two FDA-approved tests. There is no collection device, including tampons and collection brushes and brooms, which is FDA approved for self-collection. Finally, mouthwash has been used for genetic (DNA) testing for epidemiologic studies, including those that send specimens through the mail [43]. Here, we applied it to HPV DNA rather than host genomic DNA. Nevertheless, the results of this analysis should be considered in relative rather than absolute terms since it is possible that that the methods in this study led to false positive and/or false negative results.

## Conclusions

Our data illustrated how self-collected specimens can result in significantly higher point prevalence of HPV (lower specificity), including carcinogenic HPV, than a cervical specimen. The point prevalence of HPV using self-collected specimens in this study was comparable to what was recently reported [44] in a nationally representative HPV survey using cervicovaginal lavages tested with the same HPV genotyping assay. On one hand, the self-collected cervicovaginal specimens, with the higher point prevalence, may be more representative of the total lower female genital tract burden to HPV than samples taken only from the cervix. Self-collection, despite the increased detection of carcinogenic HPV, is not as sensitive as a clinician-collection for detecting prevalent cervical precancer or cancer [6]. However, it is unknown whether added detection of vaginal carcinogenic HPV not found at the cervix is in anyway associated with future risk of the cervical or lower genital tract HPV-related cancer.

## Competing interests

Dr. Castle serves on a data and safety monitoring board for Merck. He has received HPV tests and testing for research at a reduced or no cost from Qiagen Roche Norchip, and MTM. Dr. Castle has served as a paid consultant to BD, Cepheid, and GE Healthcare and has received an honorarium for speaking from Roche.

## Authors’ contributions

PEC participated in the design and oversaw the execution of the study, conducted the primary analysis, and drafted the manuscript. JCG assisted in the analysis and the drafted the manuscript. EEP participated in the design of the study and the conduct of the study. AR participated in the design and was responsible for the clinical care of the patients. PEG did the HPV testing and helped to draft the manuscript. ICS participated in the design and oversaw the execution of the study, and helped to draft the manuscript. All authors read and approved the final manuscript.

The intramural research program of the NIH/NCI and the NCI’s Center for Reducing Cancer Health Disparities supported this work.

## Pre-publication history

The pre-publication history for this paper can be accessed here:

http://www.biomedcentral.com/1471-2334/13/5/prepub

## Supplementary Material

Additional file 1: Table S1 The prevalence of 37 HPV genotypes in clinician-collected and self-collected specimens. Bold type indicates statistically greater prevalence of that type as determined using an exact version of the McNemar’s tests. Click here for file
