# Supplementary material for: Human papillomavirus genotypes detected in clinician-collected and self-collected specimens from women living in the Mississippi Delta
Source: BMC Infect Dis. 2013 Jan 7;13:5. doi: 10.1186/1471-2334-13-5 (PMC3570306; doi:10.1186/1471-2334-13-5)
Supplement: Additional file 1: Table S1 — The prevalence of 37 HPV genotypes in clinician-collected and self-collected specimens. Bold type indicates statistically greater prevalence of that type as determined using an exact version of the McNemar’s tests. [file 1471-2334-13-5-S1.ppt]

## Slide 1
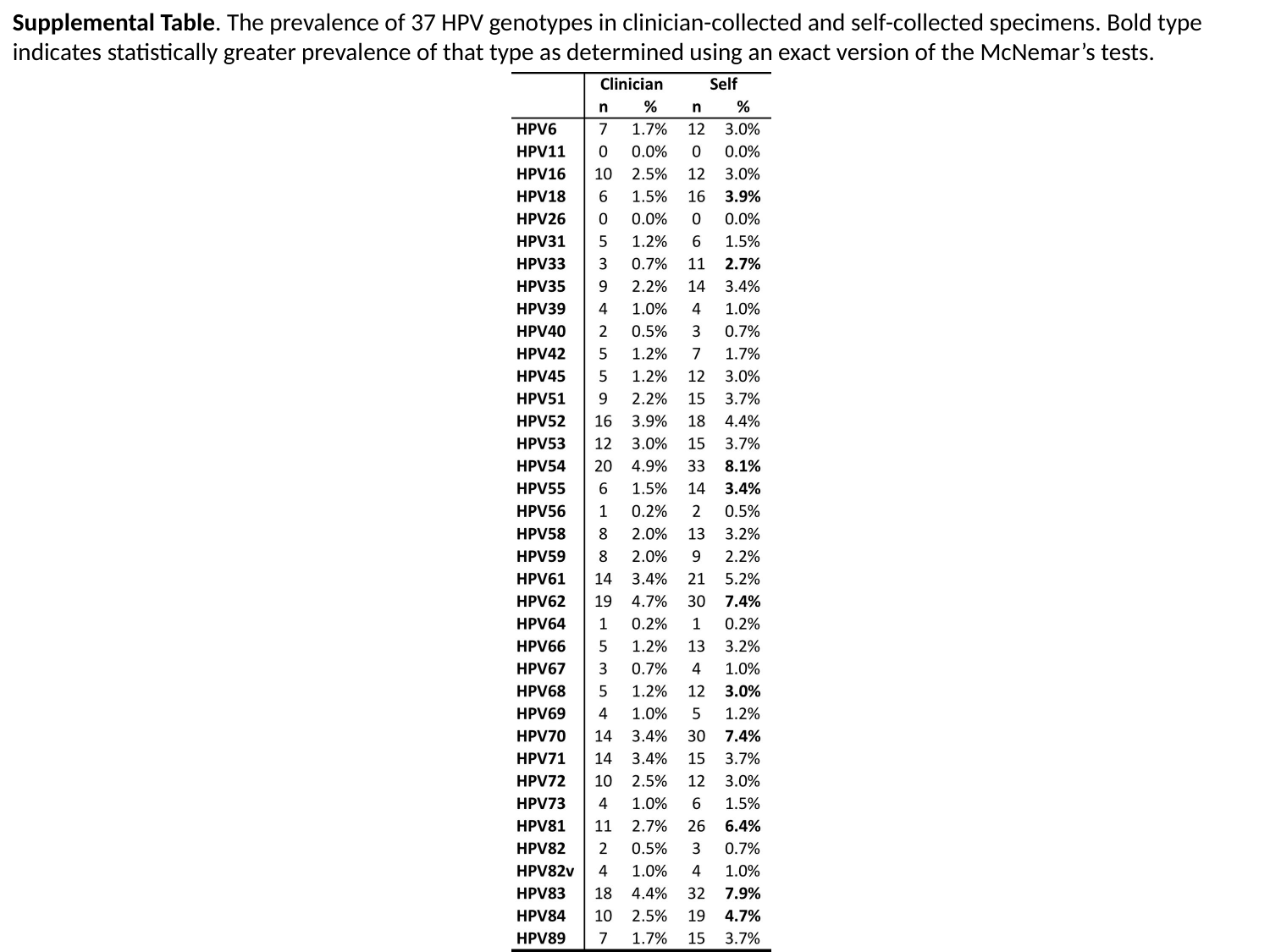

Supplemental Table. The prevalence of 37 HPV genotypes in clinician-collected and self-collected specimens. Bold type indicates statistically greater prevalence of that type as determined using an exact version of the McNemar’s tests.
